# Missed treatment opportunities and barriers to comprehensive treatment for sexual violence survivors in Kenya: a mixed methods study

**DOI:** 10.1186/s12889-018-5681-5

**Published:** 2018-06-19

**Authors:** Anne Gatuguta, Katherine G. Merrill, Manuela Colombini, Seyi Soremekun, Janet Seeley, Isaac Mwanzo, Karen Devries

**Affiliations:** 1 Department of Global Health and Development, Faculty of Public Health and Policy, London School of Hygiene and Tropical Medicine, Keppel Street, London, WC1E 7HT UK; 2 Department of Community Health, School of Public Health, Kenyatta University, Nairobi, Kenya; 3 Department of International Health, Johns Hopkins Bloomberg School of Public Health, Baltimore, MD USA; 4 Faculty of Epidemiology and Public Health, London School of Hygiene and Tropical Medicine, London, UK

**Keywords:** Sexual violence, Healthcare, HIV PEP-Kenya

## Abstract

**Background:**

In Kenya, most sexual violence survivors either do not access healthcare, access healthcare late or do not complete treatment. To design interventions that ensure optimal healthcare for survivors, it is important to understand the characteristics of those who do and do not access healthcare. In this paper, we aim to: compare the characteristics of survivors who present for healthcare to those of survivors reporting violence on national surveys; understand the healthcare services provided to survivors; and, identify barriers to treatment.

**Methods:**

A mixed methods approach was used. Hospital records for survivors from two referral hospitals were compared with national-level data from the Kenya Demographic and Health Survey 2014, and the Violence Against Children Survey 2010. Descriptive summaries were calculated and differences in characteristics of the survivors assessed using chi-square tests. Qualitative data from six in-depth interviews with healthcare providers were analysed thematically.

**Results:**

Among the 543 hospital respondents, 93.2% were female; 69.5% single; 71.9% knew the perpetrator; and 69.2% were children below 18 years. Compared to respondents disclosing sexual violence in nationally representative datasets, those who presented at hospital were less likely to be partnered, male, or assaulted by an intimate partner. Data suggest missed opportunities for treatment among those who did present to hospital: HIV PEP and other STI prophylaxis was not given to 30 and 16% of survivors respectively; 43% of eligible women did not receive emergency contraceptive; and, laboratory results were missing in more than 40% of the records. Those aged 18 years or below and those assaulted by known perpetrators were more likely to miss being put on HIV PEP. Qualitative data highlighted challenges in accessing and providing healthcare that included stigma, lack of staff training, missing equipment and poor coordination of services.

**Conclusions:**

Nationally, survivors at higher risk of not accessing healthcare include older survivors; partnered or ever partnered survivors; survivors experiencing sexual violence from intimate partners; children experiencing violence in schools; and men. Interventions at the community level should target survivors who are unlikely to access healthcare and address barriers to early access to care. Staff training and specific clinical guidelines/protocols for treating children are urgently needed.

## Background

Sexual violence is a serious global health problem with significant physical, psychological and social consequences [1–4]. The World Health Organisation (WHO) recommends that survivors of sexual violence should get immediate comprehensive treatment and be followed up for up to 6 months [5]. Immediate treatment involves attention to physical injuries, psychological trauma, prevention of unwanted pregnancy and prevention of HIV and other sexually transmitted infections (STIs) [3, 5]. Long term, survivors of sexual violence are at an increased risk of STIs, abortion, anxiety, depression, post-traumatic stress disorder, suicidal ideation and substance use [2–5]. Furthermore, experiencing sexual violence as a child is associated with negative health outcomes in adulthood including mental health problems, STIs, high risk behaviours such as having multiple sexual partners, unprotected sex, transactional sex and substance use [6–10]. Despite the significant health consequences, research has shown that the majority of survivors do not access healthcare [2–4, 11] and many of those who do, do not complete treatment [12–15].

In Kenya, sexual violence is one of the top 10 risk factors for disease burden [16]. National-level data show that 14% of women and 6% of men age 15–49 years have experienced sexual violence in their lifetime [17]. Reported national prevalence is even higher among children and young adults, with 32% of females and 18% of males reporting having experienced some form of sexual violence before the age of 18 years [18]. More than 90% of these survivors do not seek healthcare [17, 18]. Among those survivors who seek healthcare, nearly half do not complete the recommended treatment and follow up [15, 19, 20]. Additionally some survivors present to hospital but are not started on some of the recommended treatment [20].

There is limited data on the reasons why so many survivors in Kenya do not access or complete treatment and why some are not started on treatment even after presenting in hospital. A few studies indicate that factors such as limited financial and human resources; lack of training on managing sexual violence; poor coordination of services; poor referral systems; costs to survivors; stigma; and, lack of active follow may contribute [15, 19–21]. Population-level data also suggest that individual characteristics such as age, marital status, place of residence, employment status, level of education and whether one has experienced both physical and sexual violence may influence help-seeking to deal with the sexual violence from either formal or informal sources [17].

In order to appropriately target interventions to improve healthcare seeking and treatment completion, a better understanding of why many survivors do not obtain or complete treatment is required. Little is known about characteristics of survivors who are least likely to access healthcare in Kenya. Healthcare providers’ perspectives are important in understanding the gaps in care for survivors who do present for healthcare. This study therefore aimed to: 1) compare the characteristics of survivors who present for healthcare at two hospitals to those of survivors reporting violence on national surveys; and 2) understand the services provided to survivors who present for healthcare and identify barriers to treatment.

## Methods

### Setting

Kenya has a six-tiered (level 1–6) healthcare system, with lower level tiers offering basic healthcare services and more specialised services available in higher-level facilities. From September to November 2015, a review of survivor data and in-depth interviews were conducted in a level four hospital (Naivasha Sub-county Referral Hospital) and a level five hospital (Thika Level 5 Hospital). Naivasha Sub-county Hospital is situated within the Nakuru County approximately 90 km northwest of the capital city Nairobi and, Thika Level 5 Hospital is located within the Kiambu County, which borders the capital Nairobi to the east. Both facilities ran an outpatient service that attends to walk-in patients, including sexual violence survivors. The two facilities were purposively selected as they both treat a high number of sexual violence survivors. Additionally, these facilities are presumably fully equipped to offer all the services required by survivors: they have trained medical personnel, all the medicines including HIV PEP, equipped laboratories, required documents and trained counsellors. Lower-level facilities often lack one or more components of the services and therefore, these two hospitals also receive survivors referred from the lower levels.

### Design

A mixed methods approach was used. Quantitative hospital data were collected through existing hospital records and qualitative data through in-depth interviews with healthcare providers. The hospital data aimed to establish the characteristics of sexual violence survivors presenting to the facility, the treatment provided and challenges encountered by healthcare providers in the process of treating survivors. Subsequently, the hospital data were compared with population-level data from national surveys to establish if any differences existed between the profile of those survivors reporting violence nationally and those accessing care in the hospitals.

### Quantitative data

#### Hospital data

Data were abstracted from post-rape care (PRC) forms into a standardised excel sheet. The PRC form is filled in for every survivor presenting at the hospital and is used for both medical and legal purposes. It documents the socio-demographic characteristics of the survivor, the nature of the sexual violence act, where it occurred and when, who the perpetrator was, the physical examination findings on presentation, investigations done, results of the investigations, treatment given and referral to any other services [22]. A total of 543 survivor records were abstracted. Once data were abstracted, all the entries were double-checked against each PRC form filling in any missing values. Where missing values or outliers could not be resolved from the PRC form only, other sources such as the counsellor’s records and the sexual and gender-based violence (SGBV) register (contains summarised data for all survivors seen at the hospital) were used. Other missing data were extrapolated judiciously. For example, if gender was missing but there was a record of a pregnancy test or contraceptive being issued, female gender was assigned.

#### Survey data

National data were obtained from the Kenya Demographic and Health Survey (KDHS) 2014 and Violence Against Children Survey (VACS) 2010 [17, 18]. The KDHS 2014 survey interviewed both men and women 15–49 years on their lifetime and current experience of sexual violence and help seeking. The VACS 2010 is a cross- sectional household survey of 13 to 24 year old females and males designed to produce national-level estimates of the prevalence of violence against children. The survey data methods for KDHS 2014 and VACS 2010 are described fully in the KDHS 2014 and VACS 2010 reports respectively [17, 18].

#### Measures (Appendix 1)

Respondents were classified as having experienced sexual violence if reporting having ever been a victim of one or more behavioural acts of sexual violence (e.g. physically forced to have sex against will, pressured to have sex when sex was unwanted, etc.). Binary variables were used to assess respondents’ sex, whether the respondent knew the perpetrator of sexual violence, and whether or not the perpetrator was a current/previous partner. We considered age as a categorical variable. We grouped marital status according to whether the respondent was: currently married or cohabitating; previously married or cohabitating; or single. Finally, we generated a variable with six groupings to consider the place where the violence occurred: at the respondent’s own home; the perpetrator’s home; someone else’s home; while traveling by foot or roadside or bush; school; or at another location (e.g. a party, public event, etc.).

#### Statistical analysis

Analyses were performed using STATA 14. Participants with missing data were omitted from the analyses where the missing values were present. To compare the characteristics of survivors who present for healthcare at the two hospitals to those of survivors reporting violence on national surveys, we appended the following datasets: the male and female datasets from the KDHS 2014, the male and female datasets from the VACS 2010, and our hospital dataset. We restricted our analysis to participants aged 15 years and above who reported having ever experienced sexual violence (*n* = 353 hospital respondents, *n* = 1069 KDHS respondents, *n* = 611 VACS respondents). Descriptive statistics were computed and characteristics of hospital survivors, those reporting sexual violence in the KDHS and the VACS were compared using chi-square or Fisher’s exact tests as appropriate.

### Qualitative data

In-depth interviews were conducted with two trauma counsellors, two clinical officers and two nurses were selected. These healthcare professionals provided services directly to the survivors, interacted most with survivors at each of the facilities, and were involved in their follow up. At each hospital, participants were identified with the help of a nurse counsellor who is the hospital’s SGBV coordinator. Eligible participants were doctors, clinical officers (COs), nurses and counsellors. Both male and female healthcare providers were interviewed. Topic guides were used to explore healthcare providers’ views on characteristics of survivors; barriers to survivors seeking and completing care; and, challenges of providing services to survivors.

#### Interview procedures

AG conducted all the interviews in a private room within the hospital. All interviews were conducted in English. Written informed consent was obtained from all participants. All interviews were audio-recorded with permission from interviewees and lasted for about 30–60 min.

#### Data analysis

Interviews were transcribed verbatim and checked against recordings. The transcripts were then imported to NVivo 11 software. Data were analysed thematically [23]. Initial themes and codes were developed by AG based on study objectives and a literature, and were reviewed by KD. During coding, any emerging new ideas and concepts from the data not categorised previously were given new themes and codes. Once data were coded, the materials with similar codes were sorted and grouped together through thematic matrices with categories and sub-categories under the main themes.

### Ethical approval

Ethical approval was obtained prior to commencement of the study from the institutional ethics review committees of the London School of Hygiene and Tropical Medicine (Ref. 9896) and Kenyatta University (Ref. PKU/386/E32).

## Results

### Socio-demographic characteristics of survivors presenting for treatment (hospital respondents)

The socio-demographic characteristics of survivors presenting at the two hospitals are summarised in Appendix 2. The majority, 506 out of 543 (93.2%) were female. The mean age was 16.8 years, with the majority (69.5%) being children below 18 years (range 1–67 years). Among adult survivors whose marital status was documented (*n* = 177), the majority were single 123 (69.5%), followed by married 32 (18.1%), separated 8 (4.5%), widowed 7 (3.9%) and divorced 7 (3.9%).

In majority of the cases, 461 out of 530 (87%), only one perpetrator was reported. The incidence occurred in the perpetrator’s home in 183 out of 407 (45%) of the times, followed by the roadside or bush 83 (20.4%), and in survivor’s own home 71 (17.4%). Most, 387 out of 538 (71.9%), of the survivors knew the perpetrators. Neighbours 30.6%, friends 17.3% and relatives 16% were among the most common known perpetrators of violence. Cases of adolescent girls willingly engaging in sex with their boyfriends and sometimes leaving home to cohabit with them accounted for a significant proportion (15.3%) of those treated. As these girls had not reached the legal age of consent, parents brought them to the hospital as cases of statutory rape.

The age and gender of respondents in the two hospitals were similar but survivors differed in marital status, type of perpetrator, number of perpetrators and place of violence (Appendix 2).

### Socio-demographic characteristics of hospital respondents compared to survivors reporting violence in KDHS 2014 and VACS 2010

#### Gender

The proportion of men reporting violence nationally differs significantly to those seeking healthcare at the hospitals (Table 1). While only 4% of those presenting for treatment are men, 28% of the KDHS respondents and 39% of VACS respondents reporting sexual violence are men (*p* < 0.001). This translates to only one man for more than twenty women among survivors who seek healthcare, compared to nearly one man for every three women reporting violence nationally.

**Table 1:** Characteristics of participants ages 15+ reporting having ever experienced sexual violence across hospital, Violence Against Children, and Kenya Demographic and Health surveys

		Hospital Respondents	2010 VACS Respondents	2014 KDHS Respondents	*p* value
Total^a^		353 (100%)	611 (29.0%)	1069 (10.1%)	
Sex	Male	15 (4.3%)	240 (39.3%)	301 (28.2%)	< 0.001
	Female	338 (95.8%)	371 (60.7%)	768 (71.8%)	
Age (*n* = 297 hospital respondents)	15–19	169 (56.9%)	272 (44.5%)	69 (6.5%)	< 0.001*
	20–24	49 (16.5%)	339 (55.5%)	163 (15.3%)	
	25–29	39 (13.1%)	*//*	250 (23.4%)	
	30–34	20 (6.7%)	*//*	194 (18.2%)	
	35–39	6 (2.1%)	*//*	172 (16.1%)	
	40–44	6 (2.1%)	*//*	122 (11.4%)	
	45–49	3 (1.0%)	*//*	81 (7.6%)	
	50+	5 (1.7%)	*//*	18 (1.7%)	
Marital status(*n* = 338 hospital respondents; *n* = 610 VACS respondents)	Currently married/cohabitating	32 (9.5%)	184 (30.2%)	753 (70.4%)	< 0.001
	Previously married/ cohabitating	22 (6.5%)	34 (5.6%)	197 (18.4%)	
	Single	284 (84.0%)	392 (64.3%)	119 (11.1%)	
Perpetrator known? (*n* = 349 hospital respondents)	Yes	119 (34.1%)	282 (46.2%)	*//*	< 0.001**
	No	230 (65.9%)	329 (53.9%)	*//*	
Relationship of perpetrator to survivor (*n* = 291 hospital respondents; *n* = 289 VACS respondents)	Current/previous partner^b^	54 (18.6%)	147 (50.9%)	589 (55.1%)	< 0.001
	Other	237 (81.4%)	142 (49.1%)	480 (44.9%)	
Place where violence occurred *n* = 257 hospital respondents; *n* = 366 VACS respondents)	Own home	41 (16.0%)	68 (18.6%)	*//*	< 0.001**
	Perpetrator’s home	110 (42.8%)	54 (14.8%)	*//*	
	Someone else’s home	12 (4.7%)	20 (5.5%)	*//*	
	Traveling by foot/roadside/bush	65 (25.3%)	70 (19.1%)	*//*	
	School	1 (0.4%)	77 (21.0%)	*//*	
	Other	28 (10.9%)	77 (21.1%)	*//*	

*p* values assessed using chi-squared tests

*VACS* violence against children survey, *KDHS* Kenya demographic and health survey

* *p* value comparing Kenya Demographic and Health Survey respondents with hospital respondents only

**p value comparing Violence Against Children Survey respondents with hospital respondents only

^a^Represents the proportion of respondents for each dataset who report having ever experienced sexual violence

^b^For hospital respondents reporting violence by current/former partner, 41 of 54 (76%) were children below 18 years and believed that the sexual encounter with the boyfriend/girlfriend was not coerced; the parent, however, reported the encounter as coerced and took them for treatment

#### Age

The age profile of survivors reporting violence in the hospital data also contrasts sharply with national reporting of violence, suggesting that older survivors are less likely to seek healthcare compared to younger survivors. While the hospital data indicate that children 15–19 years constitute the highest proportion (57%) of survivors presenting for care, KDHS 2014 data show that adult survivors are more likely to report experience of violence (*p* < 0.001). For instance, only 6.5% of those reporting violence are 15–19 years with the highest proportion of survivors being 25–30 years (23%), followed by 30–34 years (18%) and 35–39 years (16%). Similarly, while more than 20% of women who reported violence in the KDHS 2014 are 40 years and above, less than 5% of those presenting for healthcare fall in this age group.

#### Marital status

Among the hospital respondents, never married survivors were more likely to present for treatment while currently married/cohabiting survivors were more likely to report experience of violence nationally (*p* < 0.001). Among the KDHS respondents, the proportion of never married survivors was 11%, currently married/cohabiting 70% and previously married/cohabiting 18%. In contrast, survivors presenting at hospital were overwhelmingly single (84%), compared to currently married/cohabiting (10%) and previously married/cohabiting (7%).

#### Perpetrators of violence

There were differences in the type of perpetrators reported by the national surveys respondents and hospital respondents. Hospital respondents were more likely to be assaulted by strangers (66%) compared to VACS respondents (54%) (*p* < 0.001). Current and past intimate partners were the most commonly reported perpetrators of violence among VACS and KDHS respondents but represented less than 19% of perpetrators among hospital respondents (*p* < 0.001). Notably, among hospital respondents reporting violence from intimate partners, a majority (76%) were adolescents who reported the sexual encounter as consensual but were brought to the hospital by disapproving parents as cases of statutory rape.

#### Place of violence

The place where violence occurred was reported only among hospital respondents and VACS respondents. However, not all of these respondents reported this; these data were missing among 27 and 40% of hospital and VACS respondents respectively. Among hospital respondents with data, violence occurred most commonly in the perpetrator’s house (43%), followed by while travelling on foot, by the roadside or bush (25%). Among the VACS responds, school was the most common place of violence (21%) followed by travelling on foot, roadside or bush (19.1%). Although most hospital respondents are of school-going age (15–19 years), it is surprising that violence reported to have occurred in school was very low (less than 1%) compared to the VACS.

### Quantitative findings on services provided to survivors in the hospital and barriers to treatment

Similar services were offered in the two hospitals. The services comprised of an initial medical history and physical examination; specimen collection and laboratory tests; treatment; and, counselling. The tests done included screening for STIs such as HIV, syphilis and hepatitis B; a pregnancy test; urine and high vaginal swab microscopy; haemoglobin and liver function tests. The treatment recommended includes HIV PEP, emergency contraceptive (EC), STI prophylaxis, anti-tetanus and hepatitis B vaccines as needed. In Naivasha Sub-county Hospital, the services were offered across different departments (outpatient, casualty, HIV comprehensive care centre, Youth centre, and Pharmacy) situated in separate buildings, ranging from a few metres to over 100 m apart. In Thika Level 5 Hospital, all services were provided at the HIV comprehensive care centre (except at night and weekends when services were offered in the outpatient department). The HIV comprehensive care centre comprised of two buildings adjacent to one another with different services offered in separate rooms.

#### Missing clinical examination findings and laboratory test results

Missing reports of clinical findings and laboratory tests results were common (Appendix 3). It was not clear from the records whether the absence of these reports was due to the services not being offered or due to failure to document the services. Physical examination findings were not documented in 5% of the survivors, psychological assessment in 14% and genital exam in 2%. In some cases, examination findings documented were very broad and/or uninformative and therefore unlikely to be useful in evaluating a patient clinically or as forensic evidence. For instance, in 46% of survivors, genital exam was reported only as “hymen broken” with no details to indicate whether this was a fresh injury or not and whether there were any other relevant genital findings suggestive of sexual violence. Failure to carry out laboratory investigations and/or to document results was particularly prominent. None of the laboratory tests had complete records: 40% HIV, 42.2% urinalysis, 40.3% swab microscopy, 43.3% VDRL and 50.4% Hepatitis B had no results.

#### HIV PEP, other STI prophylaxis and emergency contraception

Review of records indicated that some survivors presented to the hospitals but did not obtain certain components of the recommended treatment (Table 2). According to the national treatment guidelines, all survivors presenting within 72 h of assault and who have a significant risk of HIV exposure (such as those with oral, vaginal and anal penetration) are eligible for HIV PEP. Among all survivors presenting at the two hospitals, 30% did not receive HIV PEP. The likelihood of not being put on HIV PEP was associated with the age of survivor (*p* < 0.001), whether the perpetrator was known or unknown (*p* < 0.001) and the relationship of the survivor to the perpetrator (*p* = 0.002). Children aged 18 years and below were more likely to miss being put on HIV PEP (37%) compared to survivors above 18 years (14%). Survivors where the perpetrator was known were also more likely to miss being put on HIV PEP (38%) compared to survivors where the perpetrator was unknown (11%). Among those who were not put on HIV PEP, the most common reason for not being put on HIV PEP was late presentation (59%). Among children 18 years or below, 67% of those who did not get HIV PEP presented late. Survivors where the perpetrator was known were also more likely to miss HIV PEP because of late presentation (62%) compared to survivors where the perpetrator was unknown (27%). Notably, 24% of all survivors who did not get HIV PEP presented within 72 h and no reasons were documented for not starting them on HIV PEP.

**Table 2:** Treatment provided to survivors presenting at hospitals by age, gender, marital status and perpetrators of violence

Survivor characteristics (*n* = 543^a^)		Treatment provided to survivors										
		HIV PEP given		Reason why HIV PEP was not given					STI prophylaxis given		EC given	
		Yes	No	Late	Known HIV-infected	Boyfriend/denies sex	PEP not indicated^b^	No reason given	Yes	No	Yes	No
All survivors		361 (69.7)	157 (30.3)	92 (58.6)	3 (1.9)	9 (5.7)	15 (9.6)	38 (24.2)	406 (83.7)	79 (16.3)	209 (56.8)	159 (43.2)
Age	</= 18 years	201 (63.2)	117 (36.8)	78 (66.7)	1 (0.8)	7 (6.0)	12 (10.3)	19 (16.2)	247 (81.5)	56 (18.5)	109 (56.2)	85 (43.8)
	> 18 years	126 (85.7)	21 (14.3)	6 (28.6)	1 (4.8)	2 (9.5)	1 (4.8)	11 (52.4)	117 (88.6)	15 (11.4)	79 (61.7)	49 (38.3)
	*P* value	< 0.001		0.001					0.065		0.324	
Gender	Male	23 (67.6)	11 (32.4)	8 (72.7)	0	0	0	3 (27.3)	22 (75.9)	7 (24.1)	N/A	N/A
	Female	338 (69.8)	146 (30.2)	84 (57.5)	3 (2.1)	9 (6.2)	15 (10.3)	35 (23.9)	22 (75.9)	7 (24.1)	209 (56.8)	159 (43.2)
	*P* value	0.788		0.819					0.238			
Marital status	Single	96 (80.7)	23 (19.3)	8 (34.8)	3 (13.0)	1 (4.4)	1 (4.4)	10 (43.5)	91 (83.5)	18 (16.5)	69 (65.1)	37 (34.9)
	Married	28 (87.5)	4 (12.5)	0	0	1 (25.0)	1 (25.0)	2 (50.0)	25 (83.3)	5 (16.7)	9 (33.3)	18 (66.7)
	Divorced/separated/widowed	17 (81.0)	4 (19.0)	1 (25.0)	0	0	2 (50.0)	1 (25.0)	16 (88.9)	2 (11.1)	8 (44.4)	10 (55.6)
	*P* value	0.401		0.173					0.869		0.027	
Type of perpetrator	Known	230 (62.0)	141 (38.0)	88 (62.4)	2 (1.4)	9 (6.4)	14 (9.9)	28 (19.9)	283 (80.6)	68 (19.4)	129 (51.2)	123 (48.8)
	Unknown	128 (89.5)	15 (10.5)	4 (26.7)	1 (6.7)	0	1 (6.7)	9 (60.0)	121 (92.4)	10 (7.6)	79 (69.3)	35 (30.7)
	*P* value	< 0.001		0.006					0.002		0.001	
Relationship to perpetrator	Relative^c^	21 (44.7)	26 (55.3)	18 (69.2)	0	0	0	8 (30.8)	32 (74.4)	11 (25.6)	8 (30.8)	18 (69.2)
	Neighbour	61 (65.6)	32 (34.4)	18 (56.3)	1 (3.1)	0	7 (21.9)	6 (18.7)	67 (78.8)	18 (21.2)	26 (50.0)	26 (50.0)
	Friend^d^	38 (74.5)	13 (25.5)	8 (61.5)	0	0	1 (7.7)	4 (30.8)	45 (90.0)	5 (10.0)	26 (70.3)	11 (29.7)
	Boyfriend^e^	18 (40)	27 (60.0)	16 (59.3)	0	7 (25.9)	4 (14.8)	0	31 (73.8)	11 (26.2)	15 (33.3)	30 (66.7)
	Current/former husband	7 (77.8)	2 (22.2)	0	0	1 (50.0)	0	1 (50.0)	6 (66.7)	3 (33.3)	5 (55.6)	4 (44.5)
	Other^f^	31 (57.4)	23 (42.6)	15 (65.2)	0	0	1 (4.4)	7 (30.4)	47 (87.0)	7 (13.0)	22 (64.7)	12 (35.3)
	*P* value	0.002		< 0.001					0.166		0.003	

*HIV PEP* HIV post exposure prophylaxis, *EC* Emergency contraceptive

^a^ Number of survivors analysed for different treatment given varies due to missing data

^b^Reasons for HIV PEP not indicated included non-penetrative violence, condom used

^c^Relative includes fathers, uncles and cousins

^d^ Friend includes non-intimate friends and acquaintances such as classmates, co-workers

^e^ Boyfriend: Intimate partners to children below 18 years who were in consensual relationships which are not sanctioned by parents therefore brought for treatment as sexual violence

^f^ Others: Includes teachers, priests, boda boda (motorcycle) taxi drivers, watchmen etc.

Prophylaxis for other sexually transmitted infections was given in 84% of all survivors. Other than whether a perpetrator was known or unknown (*p* = 0.002), no other survivor characteristics were associated with being put on STI prophylaxis. Among women whose records documented whether EC was given or not, 57% received EC. Single women were more likely to receive EC (*p* = 0.027) compared to previously partnered or married women. Similarly, women where the perpetrator was unknown were more likely to receive EC compared to women where the perpetrator was known (*p* = 0.001).

### Qualitative findings on socio-demographic characteristics of survivors

Healthcare providers’ (HCPs) views expressed during the interviews helped to explain some of the observed differences between survivors presenting at the hospitals and those reporting violence nationally.

#### Stigma influences the type of survivors seeking healthcare

Sexual violence stigma influenced the type of survivors presenting at the hospitals. According to HCPs, men were under-represented among the survivors treated compared to the numbers in the community as men were afraid of identifying as survivors at the community level. The HCPs observed that stigma at the community level also affected adult females. A culture of blaming women for provoking the violence through dressing skimpily, being in the wrong place or through their work prevented them from disclosing and seeking treatment.

“They [men survivors] are there but they do not come. Because, there is that stigmatisation and that fear of how the community is going to portray you.” (Professional healthcare provider, HCP3)

“Ok some people relate it [violence] with your job. Probably you are a bar maid, so even if you are raped they usually take it that you are the one who provoked the perpetrator. And then again, also some in the community, how you dress. They tell you now you are the one who attracted the perpetrator to you.” (Professional healthcare provider, HCP5)

Healthcare providers reported that most of the survivors were from disadvantaged and poor backgrounds. They were also more likely to come from particular communities within the hospital’s catchment area. The fact that survivors were more likely to come from certain areas could have implications on sexual violence prevention. Cultural norms and the status of women in society were reported as predisposing factors to sexual violence in these areas. Survivors from such areas were likely to present for treatment only after multiple incidents of violence and many other cases in such areas were unreported.

“Let me say the community that lives there [an area where many of the survivors come from], the men, they don’t value so much a woman according to what they tell me. They consider a young person, a young woman, a young girl as a wife. So anytime there is a case that happens, when they come, they report that it’s something that has been going on for a while (…). The other reason is they (the women) are also not learned. They don’t have the knowledge of their sexual and reproductive health rights.” **(**Professional healthcare provider, HCP3**)**

HCPs therefore highlighted the importance of community awareness in improving healthcare seeking and follow-up. There was reported lack of general awareness on sexual violence in the community and lack of awareness of available services and where to find them. The lack of awareness is also compounded by the level of stigma in certain communities. HCPs observed that awareness creation would enable survivors to present for care by reducing community stigma and also being aware of the available services and the importance of seeking and completing treatment.

“The awareness needs to be raised so that people don’t feel that it’s awkward to go to the hospital when you’re sexually assaulted. Because, in as much as I’ve said it’s not there [stigma], there is still some component of it in the village, especially in the village because those who come directly, most of them are from the town […] So, campaigns to raise awareness through the community health workers, something of the sort.” (Professional healthcare provider, HCP1)

#### Types of perpetrators

The profile of perpetrators as reported by healthcare providers mirrored that observed among hospital respondents’ quantitative data. Interviewed healthcare providers elaborated on the kind of perpetrators reported and the circumstances in which violence took place. Reported perpetrators were mostly well known particularly in children and adolescents. Grooming in young children was reported as the main reason why children did not report violence as the perpetrators enticed children with gifts often building a relationship over time before the actual sexual act. In contrast, adolescents were often taken advantage of in circumstances where they had diminished capacity to make decisions.

“They (perpetrators) are of course people well known especially to the children and even I have realised, even to adults. Occasionally you will get those who do not purely know the survivors, but especially in adolescents, majority of them they know the survivors, they are their friends, they go for parties in company of others, when they are drunk, they are taken advantage of (…). Even in children because when they come, those ones of a young age, they will talk of uncle or even a relative and not uncle in quotes but a real uncle. Others step fathers, occasionally even a father.” (Professional healthcare provider, HCP4)

Quantitative findings indicated that some adolescents were treated as sexual violence survivors even though they reported being in consensual relationships, sometimes even cohabiting with the alleged perpetrators. During follow up informal discussions to clarify this, HCPs observed that some of the adolescents were brought to hospital by parents/guardians who did not approve of these relationships. As the adolescents had not reached the legal age of consent, they were brought as cases of statutory rape. Parents could then use evidence obtained from hospital to prosecute the alleged perpetrator and stop the relationship.

### Qualitative findings on barriers to accessing and receiving quality treatment

Interviewed HCPs provided some insight to the process of getting treatment once at the hospital, reasons as to why some expected treatment might not have been given as well as challenges associated with providing treatment.

#### Late presentation

HCPs reported that survivors gave various reasons for presenting late—the most common reason for not getting HIV PEP—such as distance, cost of transport to get to the hospital, fear of disclosure and threats from the perpetrator. Late presentation was particularly common in children, as children often did not disclose the abuse immediately due to fear and abuse was often discovered after complications set in.

“The main challenge is the children. Because you see most of the times after the ordeal has happened, these children are threatened. They don’t talk about it until maybe much later when the parent or the guardian may realise there is a problem, or maybe immediately the guardian may realise am seeing some funny behaviours or I can see there is something wrong with my child and they try to peruse and they will come out with the information. But if not very severely damaged, these children will keep quiet. You know most of the times, they say they are enticed, they are given gifts, they are given biscuits and all that. So they don’t really talk about it […]. So that is why some of these children will come late and they don’t get the services as required.” (Professional healthcare provider, HCP4)

#### Poor coordination of services

Facility-related challenges were also identified that hindered efficient treatment of survivors once at the hospital. Survivors being attended to at different service delivery points, in different buildings resulted not only in delays but also survivors being lost along the in-hospital pathways.

**Interviewer:** Are there situations where they don’t get there (counseling department)?

**Respondent:** “Yes there are situations. Because, the ones who don’t go… are the ones who have come and we [clinicians] have seen them, have filled the forms, but have been kept waiting for long hours in the outpatient department. They feel frustrated. And they have that mentality that if I go to the other centres am being sent to, I will still wait the way I have just been waiting in the outpatient department.” (Professional healthcare provider, HCP3)

Despite the fact that there was hospital policy that survivors should be treated as soon as possible without waiting in queues, survivors were still kept waiting for a long time in some service delivery points. Various reasons contributed to survivors waiting and included clinicians being busy with other patients and delays in some departments.

“Timeliness is still an issue. Because, we are really not seeing them with the speed that we would like to see them with. Maybe delays have been caused, maybe the survivor is supposed to see a clinical officer and maybe the clinical officer is attending to another patient […]. We are also seeing delays sometimes in the lab.” (Professional healthcare provider, HCP2)

#### Lack of equipment

Lack of necessary equipment to carry out comprehensive management such as speculums and rape kits was also a hindrance to survivor’s care. Furthermore, there were occasions when HIV test kits were missing and survivors were forced to buy them. This could perhaps explain the fact that 40% of survivor records did not have swab and HIV results. The lack of equipment limited the quality of care that could be provided to survivors. Lack of speculums, for instance, limited the ability of clinicians to collect quality specimens necessary for both treatment and forensic evidence.

“Sometimes we lack even the kits [HIV test], like now it’s only me who has them. I only have a few, so if they want to test like now this lady who was here [another counsellor] they come for them […] sometimes we don’t have. […] We tell them [survivors] to do it outside and come with results.” (Professional healthcare provider, HCP6)

“Well, challenge with speculums, they aren’t enough. There are times you may go for a speculum and you are told right now we don’t have. So you’ll keep the patient waiting and, some clinician’s do it (high vaginal swab) without, which really should not be the case. It’s a malpractice because if you do it without a speculum, you are likely to even introduce more infections and the results also can be affected by that.” (Professional healthcare provider, HCP2)

#### Few trained HCPs

Limited training was noted as a major hindrance to providing quality services to survivors. In two departments at the forefront of survivor’s care, HCPs estimated that only about three of approximately 13 staff working in the department were trained to handle survivors. Moreover, although children were the most common survivors, none of the HCPs had training on dealing with child survivors. This shortage limited the number of staff members who were confident in managing the survivors, impacting negatively on the treatment of survivors. In some instances, survivors had to wait for a particular individual to receive certain services.

“I think what we can do is, one, we need CMEs (continuous medical education) and workshops. At least all health workers should know what to do to such clients because if we all depend on [name of colleague] that she is the one who has done sexual based violence, she can be able to do 1, 2, 3... It will be quite hard, because some people- right now, at least I am better off because I went for a workshop once for gender-based violence. But you see some people have not, and you know I also won’t be at work throughout.” (Professional healthcare provider, HCP5)

#### Lack of follow up

The hospitals did not have a formal way of following up survivors to ensure that they returned to complete their treatment. Healthcare providers estimated that only about 30–50% of survivors came back after the initial treatment. The HCPs noted that they were limited in their ability to carry out the role of following up survivors. Instead, this would only be possible through involving other players at the community level such as community health workers.

“As a clinician I think it would only be possible [to follow up] if the community is involved. Because as a clinician I don’t think I can be able to follow up anyone unless I have special interests, unless I want to. Because even the other patients the general patients, I don’t get to know what happened to them unless I have a special interest.” (Professional healthcare provider, HCP1)

## Discussion

Three key findings emerged from this study: 1) Certain groups of survivors reporting violence nationally are unequally represented in seeking treatment; 2) within health facilities, various gaps exist leading survivors to miss out on essential treatment even after presenting for care; and, 3) while children below 18 years form a disproportionately large proportion of survivors presenting to hospitals, there are exceptional limitations to children’s services.

### Discrepancies in survivors reporting violence nationally and those presenting for treatment in hospital

Although national survey data show that reporting of violence increases with age and older survivors are more likely to report sexual violence [17], our hospital data seems to contradicts this. Together with multiple other studies in Kenya [15, 19–21, 24], our data show that children are more likely to present for treatment in hospitals. Moreover, the proportion of male survivors presenting for care is low compared to the proportion reporting violence, indicating that the vast majority of men who experience sexual violence do not seek healthcare. The reasons why older female survivors and men in Kenya do not access healthcare are not clearly documented, however, our qualitative data from HCPs suggests that stigma is a significant reason. Studies on healthcare seeking for sexual violence, especially in men, in the African setting are limited. Our findings correspond to a study done in the Democratic Republic of Congo that similarly identified stigma and shame as well as other barriers such as costs of care and limited availability of services as contributing factors to men missing out on treatment [25]. It is possible that the higher reporting in older women and men but less treatment seeking may be due to adults reporting incidences of violence that occurred during their childhood. However, the fact that the national survey also shows that most survivors are currently married/cohabiting and the majority are assaulted by current/previous intimate partners- incidences that likely occurred in adulthood- suggests that the low numbers of adults in hospital are more likely due to failure to seek treatment. More studies in the local context; efforts to identify those affected but not receiving treatment; and, targeted services for men are needed.

The current quantitative data also suggest that survivors experiencing intimate partner sexual violence may not be seeking healthcare in most cases. Population-level studies consistently indicate that the most common perpetrators of forced sex in both women and men are intimate partners [17, 26, 27]. The low number of survivors of intimate partner sexual violence seeking healthcare in Kenya is not surprising given the pervasive culture of acceptance of partner violence among women, stigma and a legal framework that does not recognise marital rape as a crime [28–31]. Nevertheless, the apparent high levels of survivors of intimate partner sexual violence not accessing healthcare is of immense concern when considered in light of health consequences, such as HIV transmission. A Kenya National AIDS Control Council report indicates that majority of new HIV infections occur among married or cohabiting couples [32]. Moreover, many of the HIV-infected couples are in discordant relationships where only one partner is infected [33, 34]. With forced sex, survivors in such relationships are at a heightened risk of HIV infection. Overall, evidence shows that IPV increases the risk of HIV acquisition among women by 50%, not only through forced sex but also other indirect pathways [35]. Specific efforts to reach these survivors should explore alternative and culturally acceptable interventions that address the community norms while providing the necessary information and linkages to care. Notably, while IPV is likely to be a chronic problem and involve different forms of violence, the services currently available at the hospitals are set up to respond to acute cases of sexual violence and no guidelines exist on dealing with IPV.

### Gaps in healthcare services

The HIV PEP initiation rate in our study (70%) is consistent with previous studies that have documented rates of HIV PEP initiation ranging from 63 to 94% [15, 19, 20, 24]. However, the fact that 24% of survivors presented to the hospital within the recommended 72 h but were not put on HIV PEP is of great concern. The reasons for not being put on HIV PEP are unclear and require further investigation. Kenya is a country with high (5.6%) HIV prevalence [34] and sexual violence represents a significant risk factor for HIV acquisition [36]. Hence, the need to start all survivors with potential exposure to HIV on prophylaxis is clear. It was notable that not being put on treatment for HIV PEP and other STIs was associated with certain survivor characteristics such as age, whether the perpetrator was known or unknown, and the perpetrator relationship to the survivor. While these findings may suggest that healthcare providers may be considering these characteristics when prescribing treatment, the national guidelines do not recommend using survivor characteristics as determinants for or against treatment. It is also possible that more treatments were administered than were documented. Not documenting treatment can compromise effective treatment as there are multiple departments involved. Additionally, not documenting other services rendered such as laboratory investigations may compromise treatment as well as effective follow-up. These findings therefore point to an area that needs to be urgently addressed in order to ensure that all survivors receive the recommended treatment.

Qualitative data indicates a need to streamline services within the hospital and for capacity building of HCPs. Very few HCPs interviewed had received any form of training on sexual violence care. WHO recommends that all healthcare providers attending to survivors should receive training not only on the clinical management of survivors, but also on relevant legal and policy guidelines as well as ethical issues such as confidentiality and reporting requirements [5]. Data also showed incomplete documentation of clinical findings, laboratory investigations and treatment was common. Poor documentation is detrimental to both the clinical management of the survivor and for forensic evidence. Test results are especially important in making decisions on what treatment to give, for follow up of the survivor and some as legal evidence if the case goes to court. There is an urgent need of training more providers to ensure timely and quality care.

### Limitations in services for children and adolescents

Children below 18 years constitute the vast majority of survivors being treated in the two hospitals, consistent with other studies done locally [15, 19–21, 24]. Although children constitute the highest proportion of those being treated, according to the VACS 2010 report, more than 90% of children who experience sexual violence do not access healthcare [18]. In addition, the current study shows that many of those children who presented did not get the necessary treatment because they presented much too late for some of the treatment to be effective. These findings are a major cause for concern given the potential long-term effects of sexual violence on children.

Children and adolescents constitute a majority of survivors being treated, however, there are no services tailored to children and HCPs lack training on managing children. While treatment for survivors is generally similar for adults and children, the dynamics of dealing with children and adolescents and their needs are vastly different from adults [37]. A recently published case study found that healthcare providers in Kenya lacked the basic skills necessary to deliver quality services to minors such as obtaining informed consent, maintaining confidentiality, conducting a physical examination that maintains the dignity of the child and collecting specimens [38]. Our qualitative interviews found that none of the healthcare providers in these facilities had received any training on dealing with children and adolescents. The national guidelines for treating survivors are highly adult-oriented with a few sections dedicated to the management of children. There is an urgent need to develop treatment protocols specific for children and adolescents, and to provide appropriate training to healthcare providers.

Protocols for treatment adolescents will need to address how to deal with young girls who are in consensual sexual relationships that are not socially and legally sanctioned as the girls have not reached the legal age of consent. This study indicated that more than 15% of the survivors at the hospitals fell in this category where parents disapprove of these relationships and therefore take the girls to hospital for treatment as sexual abuse. While this is clearly a legal issue, healthcare providers are faced with an ethics and rights dilemma as these survivors might be brought to hospital against their will and subjected to examination and treatment that they do not wish to receive. It is not clear whether bringing these girls to hospital and treating them as sexual violence cases actually stops the on-going relationships. More importantly, there are no guidelines for clinicians on what rationale or criteria they should use to make decisions on the type of treatment and additional support to offer.

It is also noteworthy that while Kenyan children frequently report sexual violence occurring in schools in national surveys such as the VACS, only a few of the children presenting at the hospital reported this. Child sexual violence in schools perpetrated by educators, peers and other school staff is common globally [39–41]. It is not clear why these children were heavily under-represented among the hospital respondents. Nevertheless, this points to schools being an important area for outreach- not only for primary prevention of sexual violence but also to get children who experience violence in schools accessing healthcare. Thus, creating linkages between schools and healthcare resources is crucial.

### Limitations of the study

Gaps in documentation were a main challenge for the quantitative hospital data. These gaps were inevitable, given our use of routinely collected clinical data. To overcome this challenge, multiple sources of data were used to fill in missing data. We also conducted the study at two hospitals. While these two hospitals are not representative nationally, they provide a good overview and are likely to capture a more wholesome picture of the survivors seeking treatment in Kenya than most previously-conducted research. As referral hospitals in two different counties, they attend to patients from both urban and rural settings, however, they are both located in urban centres. It is therefore likely that the profile of survivors attended to is different and sexual violence service provision differs (and is likely better) than more rural hospitals. Finally, the hospital data are not representative nationally.

The small number of HCPs interviewed limited qualitative data and therefore theoretical saturation may not have been reached. However, these HCPs were purposely chosen to represent those routinely attending to sexual violence survivors, capturing the most significant experiences and an in-depth knowledge of the issues involved.

The VACS data excludes children below 13 years and the KDHS excludes children below 15 years, therefore, no comparisons for children below 15 years were made. Additionally, while the KDHS survey asks about violence perpetrated by both intimate and non-intimate partners, information on intimate partner violence is collected in more details and the findings should be viewed with this in mind. Moreover, the study populations differed in terms of recall duration for the measures: in the surveys, we assessed survivors who had ever reported sexual violence while in the hospital data, survivors could present at any time, but it is more likely to have been recent sexual violence. Therefore, while younger people were more likely to present in the hospital data compared to older survivors in the KDHS data, this might reflect both age differences in reporting of sexual violence but may also be an indicator of how recent the violence was i.e. younger people having more recent violence and therefore presenting at the hospitals. Overall, triangulation of national survey data, quantitative and qualitative hospital data enabled our data to be more comprehensive.

## Conclusions

Our study finds that multiple barriers both at the hospital and community level result in missed treatment opportunities for survivors. Although national guidelines are available, the operationalisation of these guidelines at the hospital level is still limited by lack of staff training, poor coordination between service delivery points, lack of specific protocols for different categories of survivors as well as unavailability of basic equipment such as HIV kits, speculums and rape kits. This not only hinders provision of quality healthcare to survivors but also the collection of forensic specimen necessary for legal procedures. Specific clinical guidelines and protocols for treating children and adolescents are needed. Additionally, as not documenting treatment differs significantly from not giving the treatment, the incomplete documentation in the hospital data is a serious problem that needs to be urgently addressed.

At the community level, older survivors; partnered or ever partnered survivors; survivors experiencing sexual violence from intimate partners; children experiencing violence in schools and men are at a higher risk of not accessing healthcare. Additionally, presenting late for treatment contributes to more survivors missing out on essential treatment. Interventions at the community level should reach out to those survivors who are unlikely to seek healthcare and address barriers to early access to care.

## Appendix 1

**Table 3:** Measures generated to compare experiences with sexual violence across the Kenya Violence against Children Survey (VACS) 2010, the Kenya Demographic and Health Survey (DHS) 2014, and data collected from two hospitals in Kenya

Dataset	Questionnaire items	Coding
VACS 2010		
Any experience of sexual violence	How many times in your life… (1)…has anyone touched you in a sexual way without your consent, but did not try to force you to have sex? (2)…has anyone tried to make you have sex against your will but did not succeed? (3)…have you been physically forced to have sex against your will and sexual intercourse was completed? (4)…has someone pressured you to have sex when you did not want to and sex happened?	Coded 1 if answered ‘1 or more time’ to any of the items; 0 if answered ‘0 times’ to all items.
Marital status	Are you currently married or living with someone as if married? Have you ever been married or lived with someone as if married?	Coded 1 if married/living with someone as if married; coded 2 if formerly married/lived with someone as if married; coded 3 if answering ‘No’ to both questions.
Perpetrator known?	*For each of the four forms of sexual violence addressed (above):* Did you know any of the people who did this to you?	Coded 1 if answered ‘Yes’ for any of the forms of violence; 0 if answered ‘No’ to all forms of violence.
Relationship of perpetrator to survivor	For *each of the four forms of sexual violence addressed (above):* Were any of the people…(a) a boyfriend/romantic partner? (b) a husband? (c) a father? (d) a mother? (e) a brother? (f) a sister? (g) an uncle? (h) an aunt? (i) another male relative? (j) another female relative? (k) a teacher? (l) police? (m) military? (n) an employer? (o) a neighbour? (p) a community elder? (q) a religious leader?	Coded 1 if answered ‘Yes’ for a given perpetrator across any of the forms of violence; 0 if answered ‘No’ for a given perpetrator across all forms of violence. Perpetrator categories regrouped to align with other datasets.
Place where violence occurred	*For each of the four forms of sexual violence addressed (above):* Now think about the most recent time [this happened]? Where were you when this incident happened? (a) my home; (b) perpetrator’s home; (c) someone else’s home; (d) party; (e) public event; (f) school; (g) car/bus; (h) traveling by foot; (i) other location.	Coded 1 if answered ‘Yes’ for a given location across any of the forms of violence; 0 if answered ‘No’ for a given location across all forms of violence. Location categories regrouped to align with other datasets.
DHS 2014		
Any experience of sexual violence	(1) Does/did your (last) partner ever do any of the following things to you…(a) physically force you to have sexual intercourse with him/her even when you did not want to? (b) physically force you to perform any other sexual acts you did not want to? (c) force you with threats or in any other way to perform sexual acts you did not want to? (2) Did any previous partner physically force you to have intercourse or perform any other sexual acts against your will? (3) At any time in your life, as a child or as an adult, has anyone [other than a partner] ever forced you in any way to have sexual intercourse or perform any other sexual acts?	Coded 1 if answered ‘Yes’ to any of the items; 0 if answered ‘No’ to all items.
Marital status	Are you currently married or living with a man/woman? Are you formerly married/did you live with a man/woman [in the past]? Have you never been married/have you never lived with a man/woman?	Coded 1 if married/living with a man/woman; coded 2 if formerly married/lived with a man/woman; coded 3 if never married (i.e. single).
Relationship of perpetrator to survivor	(1) Does/did your (last) partner ever do any of the following things to you…(a) physically force you to have sexual intercourse with him/her even when you did not want to? (b) physically force you to perform any other sexual acts you did not want to? (c) force you with threats or in any other way to perform sexual acts you did not want to? (2) Did any previous partner physically force you to have intercourse or perform any other sexual acts against your will? (3) At any time in your life, as a child or as an adult, has anyone [other than a partner] ever forced you in any way to have sexual intercourse or perform any other sexual acts?	Coded 1 for “Current/previous partner” if answered ‘Yes’ to questions 1a, 1b, 1c or 2; coded 2 for “Other” if answered ‘Yes’ to question 3.
Hospital Survey		
Any experience of sexual violence	N/A *(participant included as having experienced sexual violence if a post-rape care form had been completed for the individual)*	N/A
Marital status	Specify your marital status *(open response).*	Coded 1 if married/living with someone; coded 2 if formerly married/lived with someone; coded 3 if single.
Perpetrator known?	Were the alleged perpetrators (indicate relation to victim): Unknown or Known *(check one box).*	Coded 1 if ticked “Known”; 0 if ticked “Unknown”.
Relationship of perpetrator to survivor	Does the survivor have any details on the assailant? Is the assailant known, is there any relation? *(open response)*	Coded 1 if a father/stepfather; coded 2 if a brother/stepbrother; coded 3 if a cousin; coded 4 if an uncle; coded 5 if another relative; coded 6 if an in-law; coded 7 if a neighbour; coded 8 if a friend/acquaintance/classmate; coded 9 if a family friend/friend to friend; coded 10 if a teacher; coded 11 if a stranger; coded 12 if a boyfriend/girlfriend; coded 13 if an ex-husband/ex-partner; coded 14 if a current husband/partner; coded 15 if a boyfriend but not considered coerced by the child; coded 16 if other. Perpetrator categories regrouped to align with other datasets.
Place where violence occurred	Specify place where assault occurred/where incident occurred *(open response).*	Coded 1 if in own home; coded 2 if in perpetrator’s home; coded 3 if in someone else’s home; coded 4 if at a party; coded 5 if at a public event; coded 6 if at school; coded 7 if traveling by car/bus/foot; coded 8 if another location.

## Appendix 2

**Table 4:** Socio-demographic characteristics of survivors seen at Naivasha and Thika Hospitals

		Naivasha Hospital n (%)	Thika Hospital n (%)	Total n (%)	*P* value (X^2^)
Gender	Female	313 (93.4)	193 (92.6)	506 (93.2)	0.772
	Male	22 (6.6)	15 (7.4)	37 (6.8)	
	Total	335 (100)	208 (100)	543 (100)	
Age	Mean age in years (Range)	16.4 (2–67)	17.6 (1–51)	16.8 (1–67)	t-test *P* = 0.1578, (CI 15.990, 17.656)
Age group	18 years and below	233 (75.2)	104 (58.8)	337 (69.2)	< 0.001
	Above 18 years	77 (24.8)	73 (41.2)	150 (30.8)	
Marital status	Single	54 (61.4)	69 (77.5)	123 (69.5)	0.008
	Married	21 (23.9)	11 (12.4)	32 (18.1)	
	Divorced	3 (3.4)	4 (4.5)	7 (3.9)	
	Separated	3 (4.4)	5 (5.6)	8 (4.5)	
	Widowed	7 (7.9)	0	7 (3.9)	
	Total	88 (100)	89 (100)	177 (100)	
Orphans & vulnerable children (OVC)	Yes	14 (4.4)	13 (9.1)	27 (5.9)	0.047
	No	304 (95.6)	130 (90.9)	434 (94.1)	
	Total	318 (100)	143 (100)	461 (100)	
Disability	Yes	14 (4.4)	5(3.1)	19 (3.9)	0.485
	No	308 (95.7)	159 (97.0)	467 (96.1)	
	Total	322 (100)	164 (100)	486 (100)	
Perpetrator known to survivor	Known	257 (77.2)	130 (63.4)	387 (71.9)	0.001
	Unknown	76 (22.8)	75 (36.6)	151 (28.1)	
	Total	333 (100)	205 (100)	538 (100)	
Perpetrator relationship to survivor	Relative	33 (15.6)	16 (16.7)	49 (16.0)	0.017
	Neighbour	66 (31.3)	28 (29.2)	94 (30.6)	
	Friend	38 (18.0)	15 (15.6)	53 (17.3)	
	Boyfriend (not coerced)	40 (19.0)	7 (7.3)	47 (15.3)	
	Partner or ex-partner	4 (1.9)	5 (5.2)	9 (2.9)	
	^a^Others	30 (14.2)	25 (26.0)	55 (17.9)	
	Total	211 (100)	96 (100)	307 (100)	
Number of perpetrators	1	294 (88.6)	167 (84.3)	461 (86.9)	0.053
	2	28 (8.4)	19 (9.6)	47 (8.9)	
	3	3 (0.9)	9 (4.6)	12 (2.3)	
	4 or more	7 (2.1)	3 (1.5)	10 (1.9)	
	Total	332 (100)	198 (100)	530 (100)	
Type of sexual violence	Vaginal	285 (87.2)	169 (83.3)	454 (85.7)	0.207
	Anal	23 (7.0)	17 (8.4)	40 (7.6)	
	Combined (Vaginal & Anal/ Oral)	5 (1.5)	9 (4.4)	14 (2.6)	
	Non-penetrative	14 (4.3)	8 (3.9)	22 (4.2)	
	Total	327 (100)	203 (100)	530 (100)	
Place of sexual violence	Perpetrator’s home	147 (49.3)	36 (33.0)	183 (45.0)	0.001
	By the roadside/bush	59 (19.8)	24 (22.0)	83 (20.4)	
	Survivor’s own home	55 (18.5)	16 (14.7)	71 (17.4)	
	Someone else’s home	10 (3.4)	9 (8.3)	19 (4.7)	
	School	1 (0.3)	0	1 (0.3)	
	Market place	1 (0.3)	0	1 (0.3)	
	^**b**^Others	25 (8.4)	24 (22.0)	49 (12.0)	
	Total	298 (100)	109 (100)	407 (100)	

^a^ Refers to perpetrators where only a few survivors reported each of them and include people known to the survivor through different associations such as local shopkeepers, employers and neighbourhood “motorcycle taxi” riders locally known as Boda bodas

^b^ Other places where violence occurred include inside vehicles, abandoned buildings

## Appendix 3

**Table 5:** Clinical examination and laboratory tests findings for survivors presenting at the two hospitals

		Naivasha Hospital *n* = 335 N (%)	Thika Hospital *n* = 208 N (%)	Total *n* = 543n (%)	*p* value
Clinical examination findings					
Physical exam	Normal	286 (85.4)	157 (75.5)	443 (81.6)	
	Abnormal	42 (12.5)	29 (13.9)	71 (13.1)	< 0.001
	Not documented	7 (2.1)	22 (10.6)	29 (5.3)	
Genital exam	Normal	45 (13.4)	48 (23.1)	93 (17.1)	
	Hymen broken only	173 (51.6)	79 (38.0)	252 (46.4)	
	Hymen broken with other signs	91 (27.2)	49 (23.6)	140 (25.8)	0.001
	Others e.g. anal signs	21 (6.3)	25 (12.0)	46 (8.5)	
	Not documented	5 (1.5)	7 (3.4)	12 (2.2)	
Psychological assessment	Calm/normal	278 (83)	149 (71.6)	427 (78.6)	
	Not calm/ other psychological disturbance	21 (6.3)	19 (9.1)	40 (7.4)	0.006
	Not documented	36 (10.7)	40 (19.2)	76 (14.0)	
Laboratory test results					
HIV	Negative	296 (88.4)	25 (12.0)	321 (59.1)	
	Positive	2 (0.6)	0	2 (0.4)	< 0.001
	Known HIV positive patient	2 (0.6)	1 (0.5)	3 (0.6)	
	Not documented	35 (10.6)	182 (87.5)	217 (40.0)	
Urinalysis	Normal	206 (61.5)	20 (9.6)	226 (41.6)	
	Abnormal	70 (20.9)	18 (8.7)	88 (16.2)	< 0.001
	Not documented	59 (17.6)	170 (81.7)	229 (42.2)	
Swab microscopy (high vaginal, oral or anal)	Normal	108 (32.2)	29 (13.9)	137 (25.2)	
	Abnormal	149 (44.5)	38 (18.3)	187 (34.4)	< 0.001
	Not documented	78 (23.3)	141 (67.8)	219 (40.3)	
Pregnancy test	Positive	30 (12.8)	3 (7.5)	33 (12.0)	0.339
	Negative	204 (87.2)	37 (92.5)	241 (88.0)	
	Total	234 (100)	40 (100)	274 (100)	
VDRL	Positive	2 (0.6)	0	2 (0.4)	
	Negative	296 (88.4)	10 (4.8)	306 (56.4)	< 0.001
	Not documented	37 (11.0)	198 (95.2)	235 (43.3)	
Hepatitis B surface antigen	Positive	1 (0.3)	0	1 (0.2)	< 0.001
	Negative	268 (80.0)	0	268 (49.4)	
	Not documented	66 (19.7)	208 (100)	274 (50.4)	

## Abbreviations

CO: Clinical officer
EC: Emergency contraceptive
HCP: Health care provider
HIV: Human immunodeficiency virus
KDHS: Kenya demographic and health survey
PEP: Post exposure prophylaxis
PRC: Post rape care
SGBV: Sexual and gender based violence
STI: Sexually transmitted infection
VACS: Violence against children survey
VDRL: Venereal disease research laboratory
WHO: World Health Organisation
